# The role of procoagulant phospholipids on the surface of circulating blood cells in thrombosis and haemostasis

**DOI:** 10.1098/rsob.210318

**Published:** 2022-04-20

**Authors:** Majd B. Protty, P. Vince Jenkins, Peter W. Collins, Valerie B. O'Donnell

**Affiliations:** Systems Immunity Research Institute, Cardiff University, Cardiff CF14 4XN, UK

**Keywords:** lipidomics, thrombosis, phospholipids, coagulation

## Abstract

Phospholipids (PLs) are found in all cell types and are required for structural support and cell activation signalling pathways. In resting cells, PLs are asymmetrically distributed throughout the plasma membrane with native procoagulant aminophospholipids (aPLs) being actively maintained in the inner leaflet of the membrane. Upon platelet activation, aPLs rapidly externalize to the outer leaflet and are essential for supporting the coagulation cascade by providing binding sites for factors in the cell-based model. More recent work has uncovered a role for enzymatically oxidized PLs (eoxPLs) in facilitating coagulation, working in concert with native aPLs. Despite this, the role of aPLs and eoxPLs in thrombo-inflammatory conditions, such as arterial and venous thrombosis, has not been fully elucidated. In this review, we describe the biochemical structures, distribution and regulation of aPL externalization and summarize the literature on eoxPL generation in circulating blood cells. We focus on the currently understood role of these lipids in mediating coagulation reactions *in vitro*, *in vivo* and in human thrombotic disease. Finally, we highlight gaps in our understanding in how these lipids vary in health and disease, which may place them as future therapeutic targets for the management of thrombo-inflammatory conditions.

## 1. Introduction to cellular lipids

Lipids are hydrophobic molecules found in all cell types and are required for structural support, energy storage and signalling. They are derived from dietary sources or generated endogenously within the cell and exist in several forms, including free fatty acids (FAs) and phospholipids (PLs). Each of these categories contains large numbers of lipids with distinct molecular structures and biological properties. Thus they are classified according to common functional groups, structural motifs and other differences such as FA chain length and hydrocarbon saturation [1]. Their metabolism and transport are highly regulated by cellular proteins, which include phospholipases, oxidases and lipid transporters.

FAs are the fundamental category of biological lipids and, therefore, the basic building blocks of more complex lipids. They comprise a hydrocarbon chain with a terminal carboxylic acid group and can be saturated or unsaturated depending on the number of double bonds [2,3]. Recent work by the LIPID MAPS consortium has agreed a shorthand annotation for FAs to describe the number of carbons and double bonds in a molecule [4]. For instance, stearic acid is described as FA 18:0, reflecting a fatty acid with 18 carbons and no double bonds. By contrast, arachidonic acid is described as FA 20:4, reflecting a fatty acid with 20 carbons and four double bonds, hence it is unsaturated. PLs consist of a glycerol molecule in which the three carbon atoms act as the backbone for attachment to two fatty acyl chains forming a hydrophobic tail, and a phosphate headgroup forming a hydrophilic head, providing it with an ‘amphipathic’ structure that is essential in maintaining the integrity of cell membranes [5]. More details on the subtypes and nomenclature of PLs will be provided below.

Agonist activation of circulating blood cells leads to significant changes to lipid composition and the formation of new biologically significant ‘bioactive’ lipids which play a key role in mediating signalling pathways both within the cell and with other cells [6]. In platelets, for instance, stimulation with thrombin or collagen leads to structural alterations to the membrane, including shape change, spreading and degranulation. Of relevance to this review, PLs and oxylipins (oxygenated FAs) have been demonstrated to be instrumental to inflammation, coagulation and haemostasis [1], as will be described in the sections below.

## 2. Phospholipids

**Figure 1.**
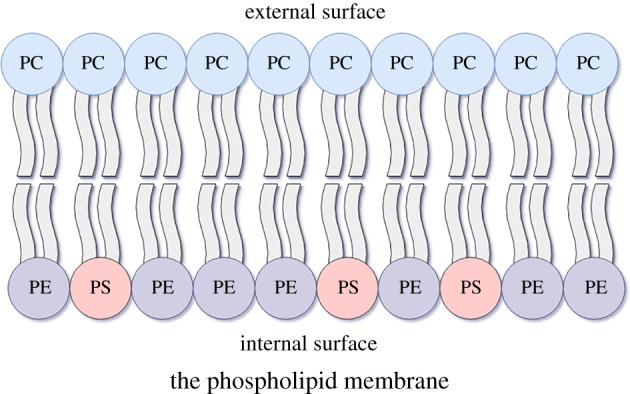
A simplified illustration of native phospholipid (PL) plasma membrane relevant to coagulation. Polarized PLs make up the membrane bilayer of all mammalian cells with phosphate head groups facing the aqueous phase and hydrophobic fatty acids facing the core. Other lipids and proteins line the membrane, which may also influence procoagulant membrane activity (e.g. sphingomyelin), but are outside the scope of this review and therefore not shown in this figure. PS, phosphatidylserine; PE, phosphatidylethanolamine; PC: phosphatidylcholine.

In common with all mammalian cells, the predominant group of structural lipids in platelets is PLs. These amphipathic lipids form the membranes of cells and organelles with the hydrophobic FA portion orientated to the core and polar phosphate-containing head groups facing the aqueous phase (figure 1) [1,5]. The resultant bi-lipid structure is described in the ‘fluid mosaic model’ of plasma membranes where they form the fluid lipid-rich phase containing a mosaic of membrane proteins [7].

**Figure 2.**
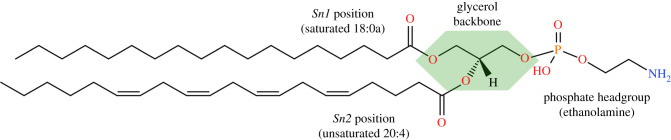
Example of a phospholipid molecule demonstrating the *sn1*/*sn2*/headgroup positions on the glycerol backbone. In this example, 1-stearoyl-2-arachidonyl-phosphatidylethanolamine, or PE 18:0a/20:4, is demonstrated with the glycerol backbone highlighted in a green polygon. Structures drawn with the aid of tools on LIPID MAPS (www.lipidmaps.org).

Glycerol forms the backbone that links the PL head groups to the FA, with the latter attaching at the *sn1* and *sn2* positions (figure 2). Generally, the *sn1* FAs are saturated or monounsaturated and can be linked to the backbone with an acyl, alkyl (ether) group or alkenyl group (plasmalogen) [8]. The *sn2* FAs are typically polyunsaturated (PUFAs) with longer acyl chains [1,5]. A combination of these variations and the different FAs can result in hundreds of unique PL species, of which the most abundant contain palmitic acid (FA 16:0), stearic acid (FA 18:0) or oleic acid (FA 18:1) at *sn1*; and linoleic acid (LA; FA 18:2), arachidonic acid (AA; FA 20:4), eicosapentaenoic acid (EPA; FA 20:5) or docosahexaenoic acid (DHA; FA 22:6) at *sn2*.

**Figure 3.**
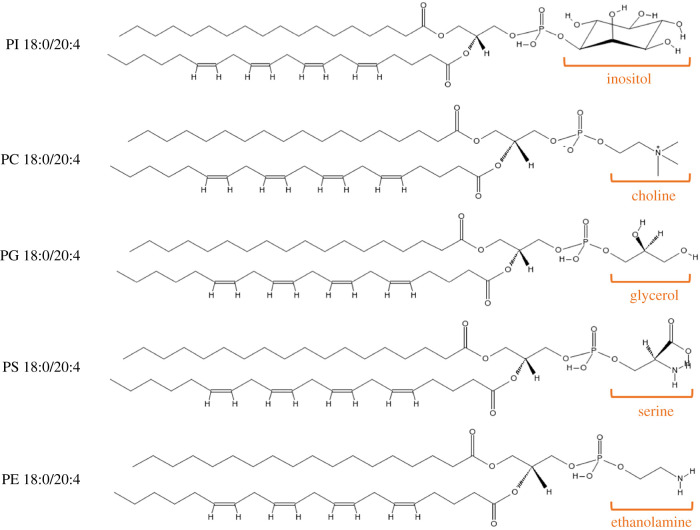
Phospholipid classes and chemical structures highlighting the phosphate head groups. In these images, the *sn1* fatty acid is stearic acid (FA 18:0) and the *sn2* fatty acid is arachidonic acid (FA 20:4). The structures of the five head groups can also be seen (PI, phosphatidylinositol; PC, phosphatidylcholine; PG, phosphatidylglycerol; PS, phosphatidylserine; PE, phosphatidylethanolamine). Structures drawn with the aid of tools on the LIPID MAPS resource (www.lipidmaps.org).

In mammalian cells, there are five main classes of PLs based on the polar head group (figure 3), specifically phosphatidylethanolamine (PE), phosphatidylcholine (PC), phosphatidylglycerol (PG), phosphatidylinositol (PI) and phosphatidylserine (PS) [5]. The most common of these are PC and PE, which together amount to approximately two-thirds of total PLs in innate immune cells [9]. Of note, AA is present in mammalian PLs with up to 10-fold higher concentrations than other PUFAs in circulating blood cells, such as platelets [5]. This is of particular relevance to this review as there is evidence that PLs with longer unsaturated FA chains support coagulation reactions somewhat better than shorter FA chains [10], as will be discussed below.

## 3. Native phospholipids and membrane asymmetry

The PL membrane of resting circulating immune cells is asymmetric, with the external leaflet being composed predominantly of PC, which also makes up 40% of total PLs. By contrast, the cytosol-facing leaflet is enriched in PE and also PS, which is present in lower amounts [11,12] (figure 1). This asymmetry of mostly neutral PLs in the outer membrane and negatively charged PS is regulated by transmembrane lipid transporters, whereby the ATP-dependent flippase or translocase keep the aminophospholipids (aPLs) PE and PS internally facing, whereas the activity of floppase, another ATP-dependent transporter, regulates the translocation of PC to the outer membrane [13].

Upon cell activation or apoptosis, PL membrane asymmetry is disrupted as a result of the rapid flux of aPLs to the outer surface. This is the result of activation of calcium-dependent scramblase, which mediates bidirectional movement of PLs [14]. Concurrently, the rise in intracellular calcium leads to the inactivation of both flippase and floppase, which halts the processes responsible for maintaining asymmetry [11,13]. The net effect is the externalization of aPLs, which alters the composition of the outer membrane and provides a net negatively charged surface. This facilitates the binding of coagulation factors on the surface, enabling generation of thrombin and fibrin (described below). Defects in this process are exemplified by Scott syndrome, a rare genetic disorder caused by a mutation to the TMEM16F scramblase protein, which causes an inability of platelets to externalize aPLs, leading to a bleeding phenotype [5,15,16]. Despite an established role in promoting thrombin formation as part of haemostasis, it remains unknown whether alterations in aPL amounts or FA composition contribute to the pathology of thrombosis, particularly since many thrombotic conditions (e.g. acute coronary syndrome) are associated with persistent thrombin formation, correlating positively with makers of inflammation such as high-sensitivity C-reactive protein (CRP) [17]. In addition, there are no pharmacological inhibitors to date which target native procoagulant PLs for the management of thrombosis in clinical practice.

## 4. The coagulation system and its interaction with aminophospholipids

**Figure 4.**
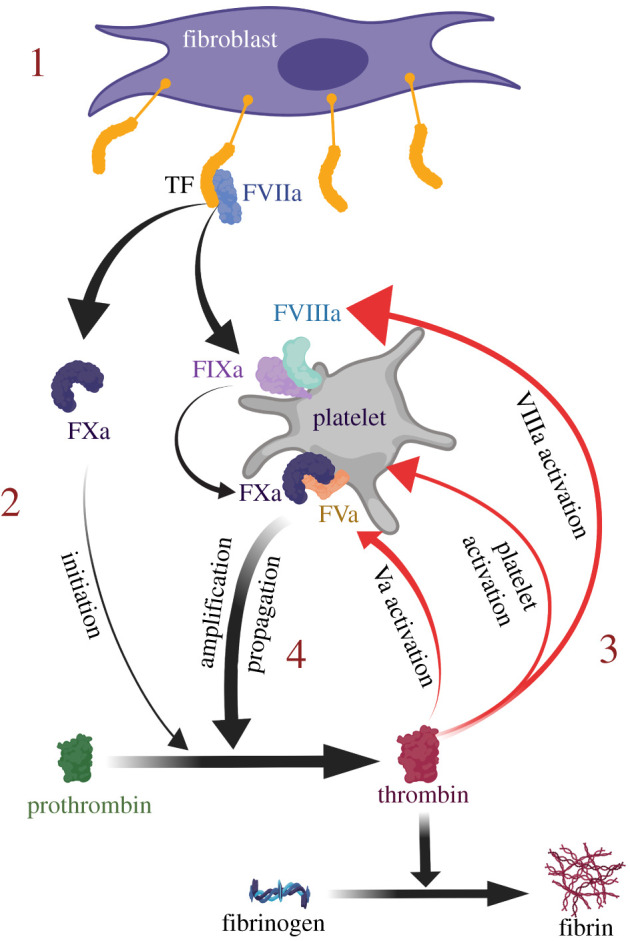
The coagulation system (cell-based model). Activation of coagulation is driven by tissue factor (TF) expressing cells in the subendothelial space (1). The TF:FVIIa complex activation of FX to FXa and FIX to FIXa is termed the ‘initiation phase’, which generates small amounts of thrombin (2). This is sufficient to activate FV to FVa and FVIII to FVIIIa, leading to the formation of FIXa:FVIIIa and FXa:FVa complexes on the platelet PL surface (3). These complexes lead to the formation of more FXa and more thrombin, respectively, as part of the ‘amplification phase’. More thrombin leads to more activated platelets and coagulation factors locally, creating a thrombin-forming ‘propagation phase’ loop (4) which leads to the formation of fibrin.

Our understanding of the coagulation system has evolved considerably from the originally described ‘coagulation cascade’ to the currently accepted ‘cell-based model’ of coagulation [18]. While both describe reactions that involve proteins known as coagulation factors, the latter model places a significant emphasis on interactions of these proteins with cell membranes. To simplify the description of this model, three overlapping phases of coagulation have been proposed: initiation, amplification and propagation [19]. These reactions require the presence of calcium ions, coagulation factors and aPLs on the external leaflet of cell membranes [20,21]. The end result of this is the formation of fibrin, which stabilizes the platelet plug and forms a clot to seal the site of the vessel injury and stop the blood loss. The different phases and contributors to this process are detailed below and are shown in figure 4.

In the initiation phase, expression of tissue factor (TF) on extravascular cells is critical for the activation of coagulation. This transmembrane protein serves as a receptor for factor VII (FVII) and its activated form (FVIIa). TF is constitutively expressed on the surface of cells surrounding blood vessels, such as smooth muscle cells and fibroblasts [22]. Disruption to vascular endothelial architecture exposes the blood to TF-expressing cells, leading to the formation of the TF:FVIIa complex, which activates the coagulation system.

Activation of TF from its encrypted to decrypted conformation may be influenced by co-expression of PS on the PL membrane, formation of disulfide bonds between cysteine residues at positions 186 and 209 and interactions with cholesterol-containing lipid rafts [21,23]. The mechanisms for this are not entirely defined but are thought to relate to interactions between PS and TF which expose the substrate binding sites for other coagulation factors [24]. These interfaces may be direct physical interactions between the polar PS headgroup and lysine residues on the TF extracellular domain [25,26], or relate to an electrostatic contact caused by the PS negative charge which may align the TF quaternary structure on the membrane surface to expose the enzymatic active site [25,26]. In both situations, the end result is activation of the TF:FVIIa extrinsic tenase complex, which initiates coagulation [27,28]. The activation of FVII to FVIIa is mediated by the presence of low levels of proteases in the circulation, including thrombin (FIIa), factor IXa (FIXa), factor Xa (FXa) and factor XIIa (FXIIa) [29–31]. Upon formation of the TF:FVIIa complex in response to vessel injury, FVII conversion to FVIIa is significantly increased by a process of autoactivation [32]. This leads to generation of more TF:FVIIa complexes which enzymatically cleave factor X to Xa (FXa) and factor IX to IXa (FIXa). Small-scale generation of FIIa takes place as a result of the action of FXa on prothrombin [33]. FXa binds to tissue factor pathway inhibitor (TFPI) and the FXa/TFPI complex inhibits the TF/FVIIa complex, terminating the initiation process (see below), after which coagulation is dependent on the amplification phase.

The amplification phase takes place on the PL surface of platelets activated by collagen and the small amount of FIIa generated in the initiation phase. Activation of factor VIII (FVIII) to FVIIIa and factor V (FV) to FVa takes place as a result of enzymatic cleavage by FIIa. These serve as cofactors for FIXa and FXa, respectively, which in turn lead to accelerated generation of FXa by the FIXa:FVIIIa ‘intrinsic tenase’ complex and of thrombin by the FXa:FVa ‘prothrombinase’ complex [19,33].

Loss of membrane asymmetry and exposure of negatively charged aPLs following platelet activation are critical to both the initiation and amplification phases as they strongly accelerate the reactions of the extrinsic tenase (TF:FVIIa), intrinsic tenase and prothrombinase complexes [34]. Exposure of the negatively charged PS leads to electrostatic and hydrophobic interactions, which increase the binding of gamma-carboxyglutamic acid-rich (GLA)-domain-containing coagulation factors (VIIa, IXa, Xa and II) to the membranes [35]. This is facilitated by calcium ions, which bind to the GLA domains and expose a hydrophobic region within the omega loop, which can then allow the coagulation factor to penetrate the PL membrane [36]. It is thought that each GLA domain has a single binding site specific for the carboxyl group on the PS headgroup as well as additional calcium-binding sites for interactions with the phosphates on any PL other than PC. Nuclear magnetic resonance (NMR) analysis has demonstrated that the PL head-group bends in order to allow its phosphate to associate with GLA-bound calcium. Lipids with a PE headgroup can interact with the GLA domains and enhance the function of PS, whereas the bulky methyl residues of the PC headgroup make it unable to participate in this process [36,37]. The increased local concentration of coagulation factors on the PL surface enhances the function and interactions of these proteins. It also facilitates transfer of substrate and product between the coagulation complexes and helps to restrict the activity of the coagulation process to areas of injury [34].

The accumulated enzyme complexes (tenase and prothrombinase) on the platelet surface support large-scale thrombin generation as part of the propagation phase [19]. This phase ensures continuous generation of thrombin and subsequently fibrin to form a sufficiently large clot. Finally, the fibrin clot is stabilized via the thrombin-mediated activation of FXIII to FXIIIa (fibrin stabilizing factor), which covalently links fibrin polymers [33].

The coagulation system is tightly regulated at various stages by a number of inhibitors which prevent inappropriate activation. TFPI is a single-chain polypeptide associated with uninjured endothelium bound to glycosaminoglycans [38,39]. It acts as a protease inhibitor blocking FVIIa and FXa activity and can also bind protein S, which enhances its anti-FXa activity [40]. Therefore, the balance between levels of TF (increased with injury) and TFPI (bound to uninjured endothelium) regulates the initiation phase of coagulation [19]. Healthy endothelium also expresses high levels of thrombomodulin (TM), which binds circulating thrombin and changes its specificity to prevent it from activating platelets or forming fibrin [19]. The resultant thrombin:TM complex becomes an activator of protein C, which regulates the amplification phase of coagulation alongside its cofactor protein S by proteolytic inactivation of FVa and FVIIIa [41]. Other regulators of the coagulation system include circulating inhibitors of thrombin such as anti-thrombin (ATIII) and alpha-2-macroglobulin [19]. It is worth noting that, for protein C and protein S to function, they also require the presence of a negatively charged membrane surface provided by PS externalization [42].

While outside the scope of this review, the plasma membrane includes a number of other lipids such as sphingomyelins whose presence contributes to modulating coagulation reactions. Resting TF-expressing cells contain abundant levels of sphinogomyelin in the outer leaflet of the plasma membrane which, alongside PS sequesteration to the inner leaflet, serves to maintain TF in an encrypted form [43]. Upon activation, these cells mobilize acid sphingomyelinase from the lysosomal compartment to the outer leaflet of the plasma membrane, which breaks down sphingomyelins, altering membrane structure and fluidity and releasing TF from sphingomyelin-mediated encryption [43]. These events contribute to TF activation and were covered in a recent review by Ansari *et al.* [44].

## 5. Externalized aminophospholipid species in platelets and leucocytes

Generally, aPL externalization is detected using flow cytometry techniques that rely on the use of annexin V or lactadherin to label the PS/PE headgroups [45–47]. These techniques are unable to distinguish between PS and PE, to determine their absolute quantities or to define their FA compositions. To address this, a mass spectrometry (MS)-based assay was developed to allow quantification of both external facing and total aPLs in platelets and leucocytes [48]. This assay is based on derivatizing aPL primary amines on the serine and ethanolamine headgroups using *N*-hydroxysuccinimide (NHS) biotin or sulfo-NHS biotin. These reagents allow labelling of total aPLs throughout the cell (NHS-biotin) or on the external face of the plasma membrane only (sulfo-NHS-biotin)NHS-biotin [49]. Biotinylation of aPLs leads to a mass shift of 226 a.m.u., which can be measured using a sensitive and specific liquid crystal (LC)-MS/MS method to distinguish external aPLs from internal forms.

Using this method, human platelets were demonstrated to externalize 3–4% of their total aPL pool upon activation with thrombin. These were composed of five molecular species of PE and three species of PS, with the majority containing AA at the *sn2* position [10,48]. The PE species were PE 16:0p/20:4, PE 18:0a/20:4, PE 18:0p/20:4, PE 18:1p/20:4 and PE 18:0a/18:1, whereas the PS species were PS 18:0a/18:1, PS 18:1a/18:1 and PS 18:0a/20:4. Apart from a patient with Scott syndrome whose thrombin-activated platelets had defective aPL externalization, no published studies to date have examined potential variations in aPL species on the surface of platelets and/or leucocytes in bleeding or thrombotic disorders [10,48].

The FA composition of aPLs appears to influence their procoagulant function. Specifically, coagulation activity is reduced in PE containing a shorter chain FA (14:0) compared with PE with AA at *sn2* [10]. Similarly, replacing AA with docosahexanoic acid (DHA) at *sn2* reduced procoagulant activity [10]. The influence of FAs on PE procoagulant activity may relate to differential interaction with coagulation factors through their GLA domains driven by the length of the FA chain [10,50]. Overall, these findings highlight the importance of FA characterization in studies investigating the procoagulant activity of aPLs, and suggest that simply examining aPL headgroup externalization (e.g. with annexin V) is insufficient.

## 6. Oxylipin generation in innate immune cells

**Figure 5.**
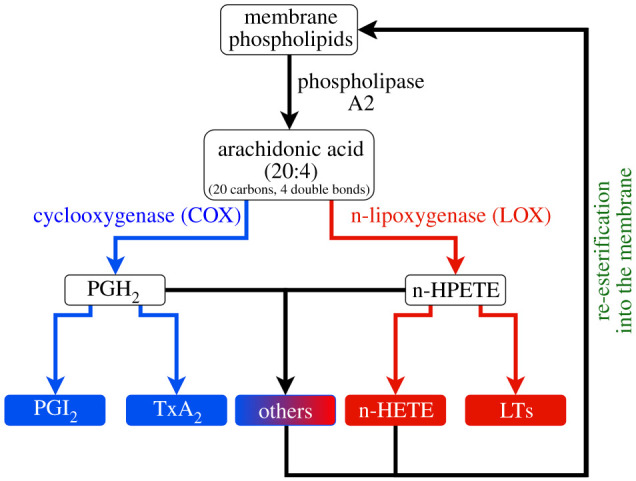
The typical oxylipin and enzymatically oxidized phospholipid pathway in circulating blood cells. Membrane phospholipids can be cleaved by phospholipase A2 (PLA_2_) into polyunsaturated fatty acids (PUFAs), such as arachidonic acid (FA 20:4). These PUFAs are oxygenated via the action of cyclooxygenase (COX) or lipoxygenase (LOX) enzymes to generate oxylipins, which are referred to as ‘eicosanoids’ if generated from 20-carbon PUFAs such as arachidonic acid (AA). Some oxylipins may be re-esterified back to the membrane to form enzymatically oxidized phospholipids. The ‘n-’ prefix denotes the enzyme isoforms which are responsible for generating oxylipin positional isomers at the corresponding ‘n-’ carbon on AA (e.g. 12-LOX in platelets generating 12-hydroxyeicosatetraenoic acids, or 12-HETE). HPETE, hydroperoxyeicosatetraenoic acid; LT, leukotriene.

Activation of immune cells leads to hydrolysis of membrane PLs via the action of phospholipase enzymes, such as phospholipase A_2_ (PLA_2_). These generate free PUFAs, which can be oxygenated by one of three enzymatic pathways: cyclooxygenase (COX), lipoxygenase (LOX) or cytochrome p450 enzymes (CYP), resulting in the generation of oxylipins [1]. AA is one of the most abundant PUFA precursors for oxylipin generation in immune cells, playing a significant role in inflammation, thrombosis and haemostasis [5]. Group IV cytosolic isoforms of PLA_2_ (cPLA_2_) are highly specific for AA-containing PLs and are regulated by the mitogen-activated protein kinase (MAPK) signalling pathway [51–53]. Figure 5 depicts a simplified version of this pathway, highlighting the COX and LOX pathways.

There are two COX isoforms, the constitutively expressed COX-1 and the inflammation-inducible COX-2 [54]. Both convert AA to prostaglandin H_2_ (PGH_2_), which is then further metabolized by several cell-specific CYP enzymes. In platelets, the CYP enzyme thromboxane synthase converts PGH_2_ to thromboxane A2 (TxA_2_), which itself is a potent secondary activator of platelets [55]. It is worth noting that aspirin and non-steroidal anti-inflammatory medications (NSAIDs) work predominantly by inhibiting COXs.

**Figure 6.**
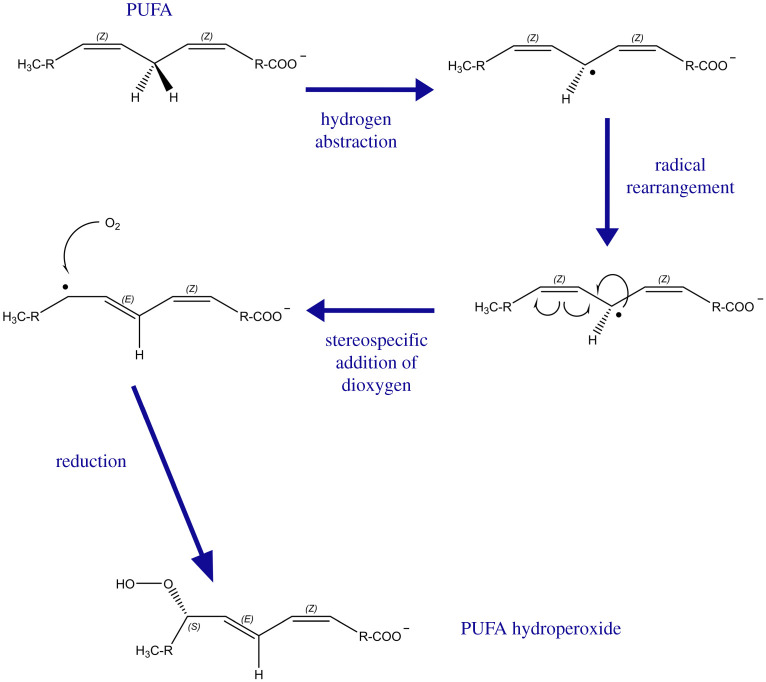
The mechanism of action of LOXs. Hydrogen atom abstraction from the PUFA substrate proceeds through a proton-coupled electron transfer (PCET) mechanism, with simultaneous transfer of electrons and protons via a concerted mechanism. Subsequently, radical rearrangement takes place with stereospecific dioxygen insertion. This is followed by a further PCET step to generate a PUFA hydroperoxide, which can then be reduced by glutathione peroxidase (GPX) enzymes to the hydroxide forms. In the case of AA, the hydroperoxides are known as HPETEs, hydroxides are known as HETEs and the position of the oxygen insertion (forming (S) stereoisomers) is dictated by the cell-specific LOX isoform. LOX, lipoxygenase, PUFA, polyunsaturated fatty acid; AA, arachidonic acid; HPETE, hydroperoxyeicosatetraenoic acid; HETE, hydroxyeicosatetraenoic acid. Adapted from Hajeyah *et al.* [58].

LOXs are a group of non-haem iron-containing enzymes which are expressed in a cell/tissue-specific manner [56]. They oxygenate AA to form hydroperoxyeicosatetraenoic acids (HPETEs), which are then reduced by glutathione peroxidases (GPXs) to form hydroxyeicosatetraenoic acids (HETEs). The oxygenation process begins with hydrogen abstraction from the PUFA, followed by radical migration and the stereospecific addition of dioxygen (figure 6) [57]. The position of the oxygen insertion is dictated by the cell-specific LOX isoform, which is named based on the most prominent oxygenation site on AA [59]. Using platelets as an example, stimulation with potent agonists such as thrombin leads to the release of AA into the cytoplasm, which is then oxygenated by 12-LOX on carbon 12 (C12) to generate 12-HPETE [60–62]. This is then rapidly reduced via GPX to 12-HETE, which may be released by activated platelets or re-esterified back to the membrane [63], as will be described below.

Other than 12-LOX in platelets, a number of other LOX enzymes are expressed in immune cells. Monocytes and neutrophils express 5-LOX, which generates 5-HPETE, a precursor of 5-HETE, and a number of leukotrienes with potent inflammatory properties [59]. There are also two 15-LOX isoforms in humans which can oxygenate AA to 15-HPETE. Of these, 15-LOX1 is found in eosinophils, reticulocytes and interleukin (IL)4/IL13-induced peripheral blood monocytes [57,64]. Unique to 15-LOX1 is the ability to directly oxygenate intact membrane PLs, as will be described below. Of specific interest to this review, no pharmacological inhibitors of LOX exist in current clinical practice, although some agents blocking target receptors for LOX products are in use, such as leukotriene receptor antagonists (e.g. montelukast) in asthma. In addition, novel LOX inhibitors are currently undergoing phase 1 clinical trials to establish their safety. An example of this is VLX-1005 (previously named ML355), which has been shown to be an effective inhibitor of 12-LOX *in vitro* and in animal studies [65]. This agent was shown to impair oxylipin generation downstream of 12-LOX and interfere with human platelet adhesion and thrombus formation at arterial shear over collagen at magnitudes comparable to aspirin and to reduce arterial thrombosis in mouse models of ferric chloride carotid artery injury [66]. However, the specificity and safety of this agent in humans remains to be tested and is the subject of a phase 1 clinical trial (NCT04783545) [67].

**Figure 7.**
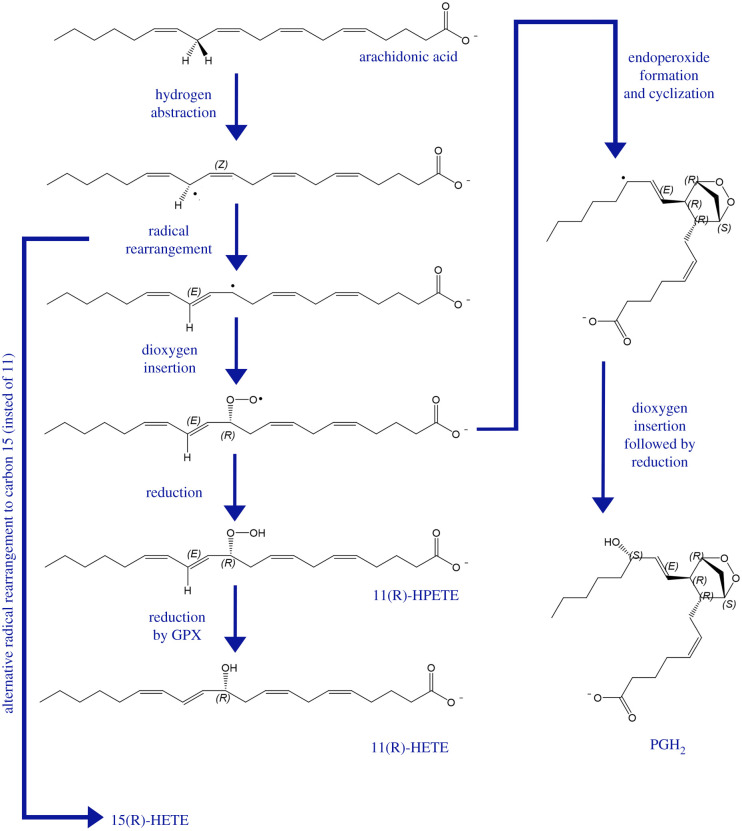
Reaction mechanism of cyclooxygenase (COX) and the generation of 11-HETE and 15-HETE as by-products of the dioxygenase reactions. Arachidonic acid (AA) undergoes hydrogen atom abstraction followed by radical rearrangement to allow for the insertion of a dioxygen molecule. The resultant product undergoes endoperoxide formation and cyclization to ultimately generate prostaglandin H2 (PGH_2_)—the intermediary product of COX-derived prostaglandins and thromboxanes. The dioxygenated AA may also undergo reduction instead of cyclization, leading to the generation of 11(R)-HPETE and subsequent reduction by glutathione peroxidase (GPX) enzymes to 11(R)-HETE. Alternative radical rearrangement to carbon 15 (instead of 11) can lead to the generation of 15(R)-HETE downstream of COX metabolism of AA. HPETE, hydroperoxyeicosatetraenoic acid; HETE, hydroxyeicosatetraenoic acid. Adapted from Hajeyah *et al.* [58].

It is worth noting that COX can also catalyse a LOX-type reaction which leads to formation of HETEs. This occurs when the COX dioxygenase activity, where one dioxygen molecule is introduced to AA, is not followed by a subsequent endoperoxide formation [68,69]. This is the result of the reduction of peroxyl radicals to form a hydroperoxide instead of undergoing cyclization. Consequently, this incomplete catalytic cycle leads to oxygenation at C11 or C15, followed by reduction, with resultant formation of 11-HETE or 15-HETE, respectively [70,71] (figure 7).

Both LOX and COX exhibit stereospecificity when it comes to oxygenating AA. This is in contrast to non-enzymatic oxygenation (e.g. by chemical oxidants) of AA, which generates equal amounts of (*S*) and (*R*) enantiomers. For LOX-generated 12-, 15- and 5-HETEs in immune cells, the hydroxyl group occurs in the *(S)* configuration, whereas COX-generated 11-HETE forms in the *(R*) configuration. Both (*S*) and (*R*) 15-HETE enantiomers can be formed by COX, but with a predominance of the (*R*) stereochemistry [70].

While this review focuses mainly on enzymatically oxidized PLs (eoxPLs) and their contribution to coagulation, it is worth noting that oxylipins (i.e. non-esterified free forms) exhibit active biological properties, including facilitating haemostatic reactions [72]. These properties are reported to vary depending on whether oxylipins are generated from omega-3 (n-3) or omega-6 (n-6) PUFAs [72]. For instance, oxylipins generated by COX-1 from AA (n-6) include thromboxane A2 (TxA2), a potent agonist of platelets which leads to platelet aggregation. By contrast, TxA3, generated by the same pathway from eicosapentaenoic acid (EPA; n-3) exhibits only slight pro-aggregatory activity [73]. In addition, DHA (n-3) metabolism by 12-LOX produces 11-hydroxy-DHA (HDHA) and 14-HDHA, which inhibit platelet reactivity downstream of the collagen receptor glycoprotein VI (GPVI), thereby reducing platelet aggregation [74]. These observations continue to drive interest in n-3 supplementation in patients at risk of cardiac disease, although utility in clinical practice is still controversial and not fully elucidated [75–77]. More on the effects of n-3 versus n-6 PUFAs and their metabolites on haemostasis has been described in a recent review by Golanski *et al.* [77].

## 7. Enzymatic formation of oxidized phospholipids

Oxylipins can be re-esterified into the membrane to form eoxPLs which alter the biophysical structure of the platelet PL surface and modify its functions [1,5]. A prime example of this is the re-esterification of HETEs back to the PL membrane to generate HETE-containing PLs (HETE-PLs). Over the last two decades, a number of HETE-PL species generated downstream of LOX enzymes have been identified following cellular activation of human immune cells including platelets [63,78,79]. These were discovered using precursor scanning electrospray ionization/tandem spectroscopy, scanning for the HETE product ion (*m/z* 319.2), thus identifying a number of precursor ions corresponding to HETE-PL species [78]. EoxPL generation will be described in more detail in sections below for both LOX and COX pathways. The role(s) of HETE-PLs is yet to be fully elucidated in health and disease, although there is an increasing body of evidence to suggest that these eoxPLs are important in mediating coagulation reactions [6]. The eoxPL synthesis pathways are described in this section with a focus on oxylipin modification and re-esterification to the PL membrane [1,5].

Following the formation of oxylipins described above, acylation with coenzyme A (CoA) may take place via the action of long-chain fatty acyl-CoA synthase (ACSL). There are numerous isoforms of ACSL, yet they are functionally differentiated by preference for specific FA chain length, tissue distribution and subcellular location [80]. Focusing on 20 carbon oxylipins (also known as eicosanoids) generated from AA, there are at least five ACSL isoforms (ACSL-1, -3, -4, -5 and -6) implicated in their conversion from FA to FA-CoA [80]. *In vitro*, Klett *et al.* [81] demonstrated that all five isoforms were able to convert HETEs to HETE-CoAs, but at differing rates and amounts. The differences in expression profiles and FA preference for individual ACSL isoenzymes enables them to channel specific FAs toward distinct metabolic fates in different tissues [81,82]. Nevertheless, studies on the specific roles of ACSL, their regulation and substrate preference in immune cells are lacking.

**Figure 8.**
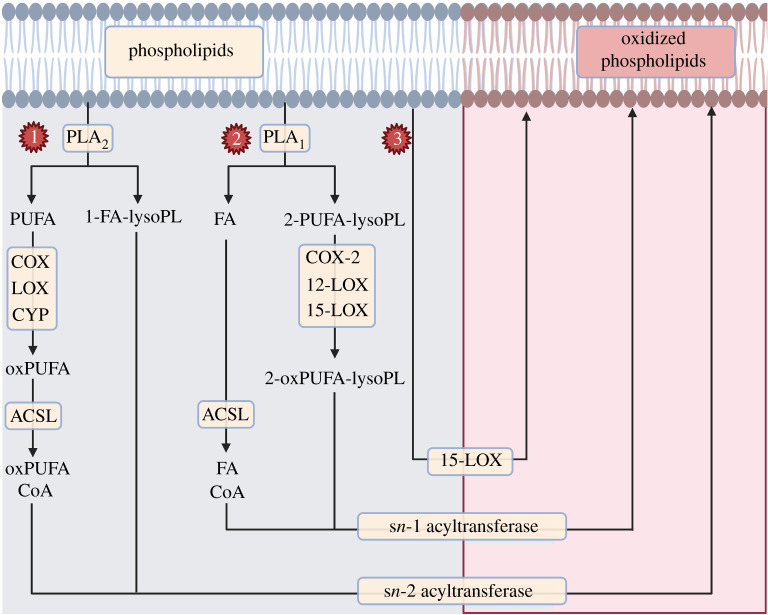
The enzymatic pathways of eoxPL biosynthesis. This figure depicts the three different pathways known to date for eoxPL generation: (1) PLA_2_ hydrolyses membrane PLs, releasing the *sn2* PUFA, which is then oxygenated by COX-11/2, LOXs or cytochrome p450 enzymes to generate oxylipins (oxPUFA). These undergo the addition of coenzyme A (CoA) via acyl-CoA synthase (ACSL), prior to being re-esterified into a lysophospholipid (lysoPL) by any one of a series of *sn2* acyltransferases (also known as LPLATs). (2) The phospholipase PLA_1_ hydrolyses membrane PLs to release 2-PUFA-lysophospholipids, which may be oxygenated by COX-2, 12S-LOX and 15-LOX prior to being re-esterified with a fatty acid CoA (FA-CoA). (3) Unique to 15-LOX is the ability to directly oxygenate membrane PL to form eoxPL.

The acyl-CoA generated downstream of ACSL may be re-esterified into a membrane lysophospholipid (lysoPL) via the action of an *sn2* acyltransferase (also known as lysophospholipid acyltransferase or LPLAT), as depicted in figure 8. This pathway is well documented in immune cells, where HETE-PL can be generated acutely on activation of neutrophils and platelets [63,78,83]. The requirement for hydrolysis and re-esterification for HETE-PL was demonstrated in both neutrophils and platelets using ^18^O-H_2_O stable isotope dilution MS and/or the LPLAT inhibitor thimerosal to block oxygenated PUFA (oxPUFA) re-esterification [63,79]. The cycle of PL hydrolysis by PLA_2_ into FA/lysoPL and subsequent re-esterification of free FA to a lysoPL by LPLATs is known as Lands' cycle and has been shown to occur in several immune cell types [6,63,79].

LPLAT enzymes vary in specificity of re-esterification substrates within Lands’ cycle. For instance, L**PC**AT demonstrates selectivity for lyso**PC** whereas L**PE**AT is selective for lyso**PE** [84,85]. Another family of LPLATs is the membrane-bound *O*-acyltransferase (MBOAT), to which the majority of LPCAT enzymes belong [86–88]. MBOATs are primarily localized within the endoplasmic reticulum and mitochondria, with 11 human genes for the different isoforms, with specific tissue distribution patterns [87]. To complicate this further, some MBOATs have a preference for specific acyl donors. For instance, MBOAT5 and -7 preferentially use AA-CoA [87,89]. Despite being selective for lysoPC, MBOAT5 (also known as LPCAT3 or LPLATA12 [90]) can esterify fatty acyl-CoAs into lysoPS and lysoPE, indicating the complexity of these enzyme cycles [91]. It is not known which of these enzyme isoforms catalyses formation of eoxPL and whether specificity varies for different oxylipins, or by the position of the hydroxyl group, in the case of HETEs.

An additional pathway for the generation of eoxPL is mediated via the action of 15-LOX, which can oxygenate membrane PLs directly without the need for PLA_2_ activity to release the PUFA [78]. This was demonstrated by Maskrey *et al.* [78] using IL4-induced monocytes incubated in buffer containing ^18^O-H_2_O and activated with calcium ionophore (A23187). In this experiment, no uptake of 15-HETE-^18^O into HETE-PEs was seen, implying direct oxygenation of PE by 15-LOX [78]. Last, recent studies showed that oxygenation of 2-AA-lysoPL by COX-2, 15-LOX2 and platelet 12-LOX forms a 2-oxylipin-lysoPL which can be converted to eoxPL via the action of *sn1* acyltransferase [92–94] (figure 8).

It is worth noting that oxPLs and oxPUFAs can be formed non-enzymatically through uncontrolled oxidation via free radical mechanisms during cell stress or inflammation. The initial reactions generate peroxyl radicals which can abstract hydrogens from a bismethylene group on PUFAs to form hydroperoxides. Intermediates formed during this process can escape to react in an uncontrolled manner, leading to non-enzymatic rearrangements of enzymatic pathway intermediates and generating a range of non-enzymatic oxPL products [95]. An example of where these processes take place in human disease is during atherosclerotic plaque formation as a result of chronic inflammation. In these plaques, a range of non-enzymatically generated oxPLs have been described with possible effects on vascular endothelial cell and macrophage functions [96–99].

## 8. Platelet enzymatically oxidized phospholipid species generated by 12-LOX

Agonist activation of platelets leads to the 12-LOX-driven synthesis of 12-HETE-PL. Studies using precursor ion scanning demonstrated the generation of four molecular species of 12-HETE-PE and two 12-HETE-PC in response to thrombin stimulation. These are PE 18:0a/12-HETE, PE 18:0p/12-HETE, PE 18:1p/12-HETE, PE 16:0p/12-HETE, PC 18:0a/12-HETE and PC 16:0a/12-HETE. They form within minutes of platelet activation and their generation is sustained for at least 3 h, mediated downstream of PAR-1 and PAR-4 receptors [63]. Furthermore, 12-HETE-PL formation required several intracellular signalling mediators such as calcium, *src* tyrosine kinase, protein kinase C (PKC) and secretory PLA_2_ (sPLA_2_) [63]. Confirming 12-LOX as the enzyme responsible for their formation, platelets from 12-LOX-deficient mice were found to be unable to generate12-HETE-PL following agonist activation [100].

Several other eoxPLs generated by 12-LOX have been reported to be generated by agonist-activated human platelets. These include 14-hydroxydocosahexaenoic acid (14-HDOHE) generated from oxidation of DHA esterified into plasmalogen (16:0p, 18:0p) or diacyl (16:0a, 18:0a) PE species [83]. These HDOHE-PE are also generated acutely by thrombin-activated platelets in the same way as 12-HETE-PL, albeit at lower levels [83]. The role of HDOHE-PL in platelets remains uncharacterized. Finally, a study from our group used targeted and untargeted lipidomic approaches to discover many previously unknown eoxPLs generated in agonist-activated platelets [101]. The most abundant of these were monohydroxy lipids derived from AA, DHA, EPA and other PUFAs. Up to now, the enzymatic pathways responsible for formation of many of these lipids remain unknown, and their structures are not fully characterized. How the platelet eoxPL lipidome varies between individuals remains unstudied.

The involvement of lipid metabolism in platelets and its impact on arterial thrombosis was further explored by a recent study from Manke *et al.* [102]. Here, the authors describe annexin A7 (ANXA7) as a regulator of oxylipin metabolism and calcium-dependent platelet activation downstream of glycoprotein VI (GPVI). Mice lacking ANAX7 or treated with its inhibitor demonstrated defective platelet aggregation downstream of collagen or the GPVI-specific agonist collagen-related peptide, translating to impaired thrombosis in a ferric chloride carotid injury model [102]. This further emphasizes the role of lipids in mediating thrombosis and their potential application as novel therapeutic targets.

## 9. Lipoxygenase-generated enzymatically oxidized phospholipid species in leucocytes

Human monocytes express 15-LOX when induced with IL4 and can, therefore, oxidize AA to 15-HETE. This enzyme is considered to be constitutively active, but can be further enhanced by stimulation of monocytes with calcium ionophore [78,103,104]. Using precursor ion scanning, 15-HETE-PL were detected in IL4-induced human monocytes, which increased upon stimulation with calcium ionophore [78]. These comprised four 15-HETE-PE species with either acyl (18:0a) or plasmalogen-linked FAs (18:0p, 18:1p, 16:0p) at the *sn1* position [78]. Furthermore, their enzymatic origin was confirmed using chiral LC, demonstrating a predominance of 15(S)-HETE attached to the PLs [78,103]. Moreover, using monocytes incubated in ^18^O-H_2_O buffer, direct oxygenation of PE by 15-LOX was demonstrated as described above [78,103]. An anti-inflammatory role for 15-HETE-PL has been proposed through binding toll-like receptor 4 (TLR4) accessory proteins such as CD14 and lipopolysaccharide-binding protein (LPB), and thus impairing the activation of TLR4 [103].

In addition to 15-HETE-PL, other 15-LOX-generated eoxPLs have been reported in ionophore-stimulated IL4-treated human monocytes. These include ketoeicosatetraenoic acid (KETE) containing PE acyl (18:0a) or plasmalogen (18:0p, 18:1p and 16:0p) lipids at the *sn1* position [105]. To confirm their enzymatic origin, studies on macrophages from 12/15-LOX-deficient mice, the murine analogue to human 15-LOX, demonstrated an absence of KETE-PE [105]. The generation of KETE-PE involves both 15-LOX and 15-hydroxyprostaglandin dehydrogenase (15-PGDH), with the latter responsible for oxidizing 15-HETE-PE to 15-KETE-PE [105].

Human neutrophils express 5-LOX, which can generate 5-HETE-PL following agonist activation with bacterial peptides, chemokines or calcium ionophore [79]. The main 5-HETE-PL species identified in neutrophils are PC 16:0a/5-HETE, PE 18:0p/5-HETE, PE 18:1p/5-HETE and PE 16:0p/5-HETE. These are generated in a coordinated mechanism involving calcium, phospholipase C (PLC), cPLA_2_ and sPLA_2_, as demonstrated by studies using pharmacological inhibitors. *In vitro* 5-HETE-PL can regulate neutrophil superoxide generation and the release of neutrophil extracellular traps [79].

## 10. Cyclooxygenase-generated enzymatically oxidized phospholipid species

COX-1 is constitutively expressed in leucocytes and platelets. By contrast, COX-2 is induced in nucleated blood cells during inflammation [54,106]. For decades, the role of COXs in generating prostaglandins and thromboxanes has been recognized [106]. Recently, however, studies demonstrated the generation of eoxPLs which contain COX-derived prostaglandins [107]. These were shown in lipid extracts from human platelets activated with thrombin, collagen or calcium ionophore analysed using precursor LC-MS/MS for eoxPLs incorporating PGE_2_ or PGD_2_. This technique demonstrated the presence of several PGE-2 PE species containing 16:0p, 18:1p, 18:0p and 18:0a at the *sn1* position. Their formation occurred within 2–5 min of platelet activation and required calcium mobilization, PLC, cPLA_2_ and *src* tyrosine kinases [107]. Aspirin supplementation (*in vivo*) and inhibitors of re-esterification (*in vitro*) inhibited their generation, indicating that they form via COX-1 activity, and downstream LPLAT-dependent esterification [107]. During identification of these lipids, other previously unknown COX-1 eoxPLs were also identified. The characterization of these lipids led to the identification of eoxPLs which contain 8,9-11,12-diepoxy-13-hydroxy-eicosadienoic acid (DiEHEDA) generated by COX-1 oxidation of AA [108,109]. These novel COX-1-derived eoxPLs use the same generation machinery described above for prostaglandin-containing eoxPLs, leading to the generation of four PE eoxPL species containing 16:0p, 18:0p, 18:1p and 18:0a at the *sn1* position. The function of these lipids is as yet unknown, but they appear to activate neutrophil integrin expression *in vitro,* which may suggest a role in modulating inflammation [110]. Finally, COX-derived 15(R)-HETE may be re-esterified into 15-HETE-PL as a by-product of incomplete cyclization, which may in turn have a role in facilitating coagulation reactions [58,94,111].

## 11. The role of enzymatically oxidized phospholipids in coagulation

In addition to native aPLs, HETE-PLs, lipids which are generated by LOX enzymes in innate immune cells, have been shown to play a role in coagulation reactions [6]. Specifically, all positional isomers of HETE-PL lead to enhanced thrombin generation *in vitro* in a dose-dependent manner [100,111]. This is thought to be related to an eoxPL-induced change in the biophysical properties of the activated cell membrane which increases the electronegativity of anionic membranes, enhancing the calcium-dependent binding of coagulation factors to the surface [100,111].

Native (unoxidized) PC cannot support coagulation factor binding, and its presence on the external leaflet ensures that the coagulation system remains inactive in resting cells. This is in contrast to 12-HETE-PC, which can directly enhance thrombin generation [11,37,100]. This is facilitated by the 12-HETE hydroxyl group, which bends up to the hydrophilic surface of the membrane and facilitates interaction with calcium, the phosphate groups of other lipids and the carboxylate group on PS [63,100]. In addition, the hydroxyl group of 12-HETE-PC may also provide increased electronegativity to the outer membrane leaflet, which may facilitate electrostatic interactions with coagulation factors [100]. Indeed, using both molecular dynamics simulations and calcium-binding assays, there is evidence that increasing the proportion of HETE-PL on liposomal surfaces increased calcium binding, which may be due to increasing electronegativity on the membrane surface [100,111].

The role of HETE-PL in supporting coagulation was examined *in vivo* using mice lacking the platelet *Alox12* gene. *Alox12^−/−^* mice generated smaller clots in response to venous injury [100]. In addition, these mice exhibited a bleeding phenotype in a tail-bleeding assay which is rescued by subcutaneously injecting 12-HETE-PL-containing liposomes into the tail [100]. Furthermore, the role of 12- and 15-HETE-PL in eosinophils has also been described as important to propagating coagulation, haemostasis and thrombotic disease [112]. This was examined in mice lacking the *Alox15* gene, which is responsible for the expression of 12/15-LOX in murine eosinophils. *Alox15^−/−^* mice demonstrated defective fibrin clot formation and reduced thrombin generation on the surface of eosinophils [112]. This defect was rescued by the addition of liposomes containing 12-HETE-PL to eosinophil mixtures, which, in addition to 15-HETE-PL, is a product of murine 12/15-LOX. The prothrombotic role of 12/15-LOX was demonstrated using a venous injury model which demonstrated smaller clot formation in *Alox15^−/−^* mice compared with wild-type controls [112]. Finally, HETE-PL were also demonstrated to play a role in murine models of abdominal aortic aneurysm (AAA), a condition that causes thrombus-containing arterial aneurysms. In these angiotensin-II-treated *ApoE^−/−^* mice, deletion of *Alox15* or *Alox12* resulted in protection against aneurysm formation. [113]. These findings suggest an *in vivo* role for HETE-PL in mediating thrombosis and haemostasis.

The role of eoxPLs in coagulation was also examined in human studies. Patients with antiphospholipid syndrome who had experienced a venous thrombotic event had higher levels of circulating eoxPLs on the surface of platelets and leucocytes as well as elevated plasma levels of immunoglobulin G that recognized eoxPLs [100]. This was observed for both platelet 12-HETE-PL as well as for leucocyte 15- and 5-HETE-PL [100]. Furthermore, patients undergoing cardio-pulmonary bypass surgery, who are known to be susceptible to elevated risk of bleeding following the procedure, had reduced platelet 12-HETE-PL post-operatively, compared with their pre-operative levels [111], with the reduction in 12-HETE-PL hypothesized to be a contributing factor to their bleeding phenotype. These findings indicate a potential association of HETE-PL with thrombosis and haemostasis.

## 12. What remains unknown about procoagulant phospholipids in thrombo-inflammation

The evidence presented above describes what is currently known about aPL distribution and eoxPL generation in response to inflammation and agonist activation of immune cells. It also discusses the role that aPLs and eoxPLs have in promoting coagulation reactions *in vitro* and in murine models of thrombosis. Nevertheless, it is not yet known how aPL and eoxPL profiles in circulating blood cells are impacted by thrombotic disease (arterial and venous) in humans. Therefore, more clinical and translational studies characterizing the procoagulant PL profiles in thrombotic conditions are needed using contemporary lipidomic techniques that rely on the use of LC-MS/MS and derivatization methods to facilitate the detection and quantification of these lipids. In addition, a mechanistic understanding of the role that these lipids play in inflammation and coagulation is needed to move the field closer towards understanding how they are modulated and modified.

In summary, characterizing the procoagulant lipidome from patients with thrombo-inflammatory conditions will improve our understanding of the role of membrane PLs in these conditions and their influence on membrane procoagulant potential. This may lead to the identification of PL-based therapeutic targets for the prevention and treatment of pathological clotting.

## Data Availability

This article has no additional data.
